# Complete genome sequence of *Pseudomonas canadensis* strain Pcan-CK-23 isolated from *Zophobas morio* larvae

**DOI:** 10.1128/mra.00023-24

**Published:** 2024-04-30

**Authors:** Cindy Kundlacz, Claudia Aldeia, Yasmine Eddoubaji, Edgar I. Campos-Madueno, Andrea Endimiani

**Affiliations:** 1 Institute for Infectious Diseases (IFIK), University of Bern, Bern, Switzerland; 2 Graduate School of Cellular and Biomedical Sciences, University of Bern, Bern, Switzerland; University of Maryland School of Medicine, Baltimore, Maryland, USA

**Keywords:** *Pseudomonas canadensis*, genome analysis, Illumina, Nanopore, *Zophobas*, larvae, *Pseudomonas extremorientalis*

## Abstract

We present the complete genome sequence of *Pseudomonas canadensis*. The strain (Pcan-CK-23) was isolated from *Zophobas morio* (superworm) larvae. The genome consisted of a 6,424,469 bp chromosome with a GC content of 60.3% and 5,973 genes. Pcan-CK-23 can be used as a reference genome for further studies with *P. canadensis*.

## ANNOUNCEMENT

Six genomes of *Pseudomonas canadensis* strains exclusively isolated from plants or soil are deposited in the NCBI database (1, 2). However, only the scaffold genome of *P. canadensis* 2–92^T^ (GenBank: GCA_000503215.1) is published (1). Here, we described the complete genome of *P. canadensis* strain Pcan-CK-23.

Pcan-CK-23 was isolated in September 2023 from homogenized tissues of four *Zophobas morio* (superworm) larvae plated onto selective ChromID ESBL plate (bioMérieux) following a 48 h incubation at room temperature (RT). Larvae were purchased from a pet shop in Switzerland during an ongoing project (https://data.snf.ch/grants/grant/206400). The larvae diet consisted of daily fresh pear pieces and dry cat food on an oat substrate for 14 days. Matrix-assisted laser desorption/ionization-time-of-flight mass spectrometry [Bruker, FlexControl v3.4 (build 135.14)] initially identified Pcan-CK-23 as *Pseudomonas extremorientalis* (score 2.02). A 20% glycerol stock was prepared from single colonies of Pcan-CK-23 and stored at −80°C.

Pcan-CK-23 was reactivated from the 20% glycerol stock followed by overnight incubation at RT on a CSBA plate (Oxoid). Genomic DNA was extracted with the PureLink microbiome DNA Purification Kit (Thermo Fisher Scientific) using multiple morphologically identical colonies. Whole-genome sequencing was achieved by combining short-read sequencing obtained with the NovaSeq 6000 instrument using the NEBNext Ultra II DNA library preparation kit for Illumina (2 × 150 bp paired-end reads; Illumina) and long-read sequencing on a MinION sequencer (Oxford Nanopore Technologies) for 48 h using the SQK-RBK114.24 rapid barcoding kit and a FLO-MIN114 R10.4.1 flow cell (reads <200 bp and Q-score of <9 were discarded). Adaptor sequences were removed from short reads using Trimmomatic v0.39, and long reads underwent preprocessing with Porechop v0.2.3 and Filtlong v0.2.1 (parameters: minimum length, 1 kb; target bases, 1 billion) to remove adaptors and further read quality filtering, respectively (3, 4). Unless otherwise indicated, default parameters were utilized for all software executions.

Sequencing generated a total of 24,696,372 short reads and 227,091 long reads (*N*_50_: 7,635 bp; mean read quality 14.3). These values correspond to the total number of unpaired reads from the raw data not preprocessed. Using the Unicycler v0.4.7 hybrid pipeline, a *de novo* and complete circular assembly of Pcan-CK-23 consisting of a chromosome (6,424,469 bp) with an average coverage of 541.3× (calculated with Qualimap) and 60.3% GC (estimated with Geneious Prime v2022.0.2) content was obtained (5, 6). The chromosome was automatically rotated by Unicycler to *repA*. The NCBI Prokaryotic Genome Annotation Pipeline (GeneMarkS-2+ v6.6) identified 5,973 genes (5,882 coding sequences; 91 RNA genes). Genome quality and reliability were estimated using CheckM v1.2.2 (parameter: lineage_wf), with 99.93% genome completeness and 0.66% contamination (7).

Type (strain) genome server (TYGS) indicated that Pcan-CK-23 was actually a *P. canadensis* strain (8). This identification was supported by average nucleotide identity with *P. canadensis* 2–92^T^ of 99.19% and 99.38% using JSpeciesWS and OrthoANIu, respectively (9). Furthermore, two complementary methods employed to identify antibiotic resistance genes, AMRFinderPlus v3.11 (parameter: minimum identity 0.6) (10) and ResFinder v4.1 (parameters: 70% threshold ID, 60% minimum length) (11), revealed the presence of *fosA8* and *bla*_PAO_ genes.

Overall, Pcan-CK-23 can be used as a reference genome for future studies with *P. canadensis*.

## Data Availability

The complete genome sequence of *P. canadensis* strain Pcan-CK-23 is deposited in GenBank under accession number CP139639.1 (BioProject accession PRJNA1047349). Sequencing reads are available in the Sequence Read Archive (SRA) under accession numbers SRR27312760 and SRR27312761 for Illumina and Nanopore reads, respectively.
